# Comorbid Psychopathology and Alcohol Use Patterns among Methadone Maintenance Treatment Patients

**DOI:** 10.1155/2015/197652

**Published:** 2015-03-23

**Authors:** Georgios Moussas, Irene Fanouraki, Argiro Pachi, Arezina Asomatou, Olga Drylli, Georgios Paschalakis, Athanasios Tselebis, Konstantinos Giotakis, Dionisios Bratis, Georgios Dermatis, Meni Malliori

**Affiliations:** ^1^Psychiatric Department, “Sotiria” General Hospital of Chest Disease, Mesogeion 152, 11527 Athens, Greece; ^2^Organization against Drugs (OKANA), Mesogeion 152, 11527 Athens, Greece; ^3^Department of Psychiatry, Athens University Medical School, Vas. Sofias 72-74, 11528 Athens, Greece

## Abstract

130 patients from a methadone maintenance treatment program agreed to complete Symptoms Checklist 90-Revised (SCL-90R) and Alcohol Use Disorders Identification Test (AUDIT) self-report scales. Scores higher than the proposed cut-score on SCL-90R scale were observed on depression, obsessions-compulsions, paranoid ideation, anxiety, anger-hostility, somatization, interpersonal sensitivity, and psychoticism subscales. In sum, 42.9% of our sample exhibited depressive symptomatology, 34.9% obsessive-compulsive symptoms, 29.1% somatization, 27.2% anxiety symptoms, 22.2% paranoid ideation, 19% phobic anxiety, 15.1% psychoticism, and 15.1% hostility and 11.9% presented with symptoms of interpersonal sensitivity. Mean score on AUDIT scale was 6.9 ± 7.9. 63.0% of our participants scored below cut-off and were classified as having a low level of alcohol-related problems; 24.4% scored in the range of 8–15 which is an indication of alcohol abuse whereas 12.6% scored 16 and above indicative of serious abuse/addiction. Scores on AUDIT scale were positively correlated with length of time on methadone treatment, but not with length of time on drug use or age of our participants. Positive correlations were observed among AUDIT and SCL-90R scores, namely, with global severity index score, positive symptom distress index, positive symptom total, and all primary symptom dimensions subscales except phobic anxiety.

## 1. Introduction

Substance abuse and dependence, especially opioid dependence, contribute significantly to the global burden of disease in Greece [1]. According to the Greek Documentation and Monitoring Centre for Drugs (EKTEPN) a rising trend in the use of all illegal drugs was observed during the last 30 years in Greece among 15–19-year-old students [2]. In 2011 the Nationwide School Population Survey on Alcohol and Other Drugs in Greece stated that 1.3% in this juvenile age group reported past heroin use [3]. The estimated number of drug users, aged 15–64 years and reporting heroin as primary drug, was 22,515 for the year 2010 in Greece [4].

Applying harm reduction policies and attempting to aid drug users who failed to benefit from abstinence “drug-free” treatment programs led to the development of the Organisation against Drugs (OKANA) in 1995, with 54 Substitution Treatment Units and a total of 9.878 persons in substitution treatment in Greece in 2012. 27.1% of individuals were in methadone and 72.9% in buprenorphine Substitution Treatment Units in 2012 [5]. During the same year 2,136 addicts requested entry to the substitution programs, while 4,275 persons were already on the waiting list since 2011 [6]. Most of them reported polysubstance abuse (74%), with heroin as a primary drug of abuse (77.5%) [5].

The high prevalence of concomitant psychiatric disorders [7–10] and alcohol abuse [11] among drug users far exceeds general population estimates and complicates the picture raising issues not extensively studied by prior international and Greek literature. While figures vary greatly, it has been estimated that between 28 and 76% of methadone maintained patients have at least one comorbid psychiatric disorder [12–14], while alcohol abuse ranges between 8 and 50% during maintenance treatment, being 10–90% before entering treatment [10].

Addiction and psychological disorders have bilateral correlation. Studies report that there are significant associations between the current measure of psychological distress on methadone maintained patients and both illegal substance use and alcohol use [15–20]. Higher comorbidity and severity of psychopathology were also correlated with family-social problems and employment status [21].

Several other studies indicate that drug-dependent subjects with current problematic substance use and coexisting psychopathology undergoing methadone maintenance require higher methadone dosages on treatment programs [22–24] tend to remain in treatment longer [25] and generally have reduced health-related quality of life [26].

Coexisting psychopathology has a further negative influence on health-related quality of life [27] and it is reasonable to assume that recognizing and treating it may also improve health-related quality of life in this population [28]. Such being the case, remaining on methadone maintenance treatment programs most likely effectuates long-term improvements in quality of life in these patients [29, 30].

Studies include psychiatric disorders as causes of addicts' rehabilitation failure [31–34]. According to literature, the more the psychopathology of drug addicts, the greater the need for therapeutic interventions [35]. Severity of psychopathology complicates treatment and subjects with less severe comorbidity respond optimally in psychotherapeutic interventions [36]. Other isolated studies [37–40] could not confirm the assumption that opiate users with psychiatric comorbidity are more vulnerable in the long-term course of addiction. Either way recent reports [41, 42] state that comorbid patients seem to have special treatment needs requiring additional psychopharmacological and psychotherapy treatments.

The present study aims at investigating the presence of psychopathological symptoms and alcohol use patterns among drug addicts receiving methadone maintenance treatment since relevant research in the Greek population has not extensively studied such issues.

## 2. Materials and Methods

### 2.1. Sample-Procedure

130 randomly selected individuals from a population list of 300 methadone maintenance patients treated in the 4th Substitution Unit of OKANA in Athens agreed to complete Symptoms Checklist 90-Revised (SCL-90R) and Alcohol Use Disorders Identification Test (AUDIT) self-report scales, during one year. Research Randomizer [43] was used to draw a random sample of participants from the population list.

Participants were informed that all study information would be kept confidential, participation (or lack thereof) would not affect their treatment, and participation was voluntary, based on verbal informed consent. All interviews were conducted by personnel separate from clinic staff in a private room at the outpatient clinic. Ethics approval was not necessary given that the nonexperimental design did not expose subjects to risks. Additionally, subjects were informed of the study's goals and procedures. Data were recorded and handled anonymously.

### 2.2. Psychological Measures

#### 2.2.1. SCL-90R

The SCL-90R is a 90-item self-report symptom inventory designed to reflect psychological symptom patterns of psychiatric and medical patients. Each item of the questionnaire is rated on a 5-point scale of distress from 0 (none) to 4 (extreme). The SCL-90R consists of the following nine primary symptom dimensions: somatization (SOM, which reflects distress arising from bodily perceptions), obsessions-compulsions (OC, which reflects obsessions-compulsions symptoms), interpersonal sensitivity (IS, which reflects feelings of personal inadequacy and inferiority in comparison with others), depression (DEP, which reflects depressive symptoms, as well as lack of motivation), anxiety (ANX, which reflects anxiety symptoms and tension), hostility (HOS, which reflects thoughts, feelings, or actions that are characteristic of negative affective states of anger, aggression, irritability, rage, and resentment), phobic anxiety (PHO, which reflects symptoms of persistent fears as responses to specific conditions), paranoid ideation (PAR, which reflects symptoms of projective thinking, hostility, suspiciousness, and fear of loss of autonomy), and psychoticism (PSY, which reflects a broad range of symptoms from mild interpersonal alienation to dramatic evidence of psychosis) [44–46].

The SCL-90 takes between 12 and 20 min to complete. With regard to its reliability, the internal consistency coefficient *α* values for the nine symptom dimensions ranged from a low of 0.77 for psychoticism to a high of 0.90 for depression. Additionally, the few validity studies of the SCL-90R demonstrate that this scale has equal validity compared with other self-report inventories. The SCL-90R has been standardized and used in the Greek population and its reliability (Cronbach's *α*) for the total of the items is 0.97 [47–50]. The cut-off for the SCL-90R subscales is 0.99 [51]. *T* scores have a normal mean of 50 and a standard deviation of 10. The cut-off level indicating clinically significant problems was set to *T* ≥ 70 [52, 53]. These are reported in the descriptive statistics for the sample.

The inventory was completed in the presence of psychologists who provided clarifications when necessary.

### 2.3. AUDIT

The Alcohol Use Disorders Identification Test (AUDIT) consists of 10 questions: three questions on use, four on dependence, and three questions about problems related to harmful alcohol use. Each response has a score ranging from 0 to 4. A total score of >8 is an indication of alcohol abuse and a score of >15 indicates serious abuse/addiction whilst a score between 8 and 10 is an indication of being at risk, according to the authors [54–57].

### 2.4. Statistical Analysis

All variables are assessed with the use of descriptive statistics and values are expressed as the mean ± standard deviation for continuous variables. Kolmogorov-Smirnov test served as a goodness of fit test indicating the normality of the distribution of our data. A one-sample *t*-test was run to determine whether AUDIT score in recruited subjects was significantly different to Greek general population, defined as AUDIT score of 3.79 [56]. Independent samples *t*-test was used to test for significance of difference between groups based on gender and employment status. One-way ANOVA was used to test for significance of difference among groups based on marital status. Pearson's correlation was used to measure the bivariate associations among continuous variables. To determine the best predictors of the dependent variable “AUDIT” we performed a stepwise multiple regression analysis with length of time on methadone treatment and (from the SCL-90R) whether or not there was psychopathology present as independent variables. Statistical significance was set at *P* < 0.05 (two-tailed). All analyses were carried out using the statistical package SPSS version 19.

## 3. Results

Of the 130 participants 98 (75.4%) were males and 32 (24.6%) females. 77 participants (59.23%) reported being single, 25 (19.23%) divorced, 23 (17.7%) married, and 5 (3.8%) widowed. 80 participants (61.5%) reported having no children whereas 50 (38.5%) of them reported having at least one. The vast majority of our sample stated being unemployed, 106 (81.53%), whereas only 24 (18.46%) reported having a job. Mean age of our sample was 43.34 ± 8.11, mean years of education were 10.34 ± 3.02, and mean length of time on drug use was 26.07 ± 7.93 years and on methadone treatment 6.69 ± 4.18 years.

In almost all subscales of the SCL-90R mean scores were >1 (Table 1). Highest mean score was observed in depressive dimension of the SCL-90R and lowest one in phobic anxiety (Table 1).

Mean AUDIT score of our sampled population (6.91 ± 7.96) was higher than the Greek general population AUDIT score of 3.79 [56], a statistically significant difference of 3.12 (95% CI, 1.73 to 4.52, *t*(126) = 4.421, and *P* < 0.0005) (Table 1).

**Figure 1:**
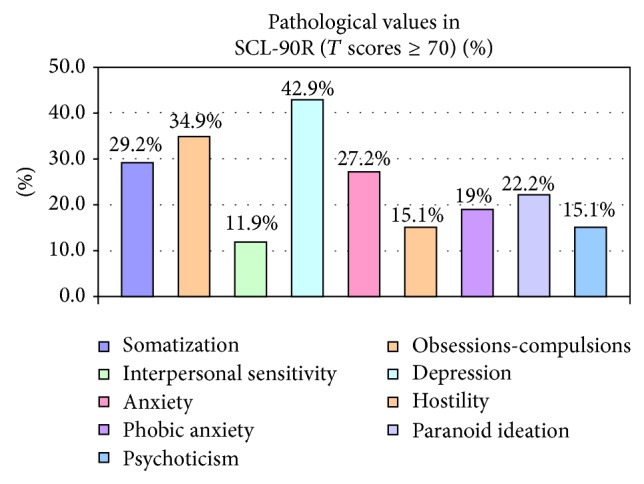


Only 6.4% of our sample scored within normal range on all nine factors of the SCL-90R scale. On the contrary, 42.9% of our participants reported symptoms over the cut-score on depressive symptomatology, whereas 34.9% presented with obsessions-compulsions symptoms (Figure 1).

24.4% of our sample scored in the range of 8–15 on AUDIT scale as an indication of alcohol abuse, and 12.6% scored >15 indicating serious abuse/addiction. 3.9% scored in the range of 16–19 indicating a high level of alcohol problems, and 8.7% scored >20 indicating possible dependence (Table 2).

As to gender the female population of our sample scored significantly higher on anxiety, hostility, depression, phobic anxiety, and somatization. Females were also significantly younger than males (40.4 versus 44.3 *P* < 0.05) and had less mean length of time on drug use (23.5 versus 27.0 *P* < 0.05) (Table 1).

Drug addicts of our sample on parenthood (*N* = 50) scored higher on depression (2.2 versus 1.8 *P* < 0.05) and somatization (1.6 versus 1.3 *P* < 0.05) compared to others without parental responsibilities.

Lower levels for anxiety (1.19 versus 1.65, *P* < 0.05) and paranoid ideation (1.37 versus 1.81, *P* < 0.05) were noted among employed individuals of our sample (*N* = 24). Divorced or separated subjects exhibited higher rates on AUDIT scale versus married ones (11.17 versus 4.52, ANOVA Bonferroni *P* < 0.05).

Age was negatively correlated with anxiety, paranoid ideation, phobic anxiety, hostility, and interpersonal sensitivity subscales (Table 3).

Education level and length of time on drug use were not correlated with psychopathology scales. A trend towards negative correlation was noted for paranoid ideation and length of time on methadone treatment.

Positive correlations were observed among scores on AUDIT scale and scores on all subscales of SCL-90R, except for phobic anxiety (Table 3). Also scores on AUDIT were positively correlated with length of time on methadone treatment.

Stepwise multiple regression analysis was conducted to identify the best predictors of the dependent variable “scores on AUDIT scale” among the independent variables “scores on subscales of SCL-90R” and “length of time on methadone treatment” and to examine their contribution to the variation (expressed as *R* ^2^) in the dependent variable. The final regression model showed that from all variables entered into the equation “somatization subscale from SCL-90R” and “years on methadone treatment” were significant predictors of “scores on AUDIT scale” (*F*_2,117_ = 8.625, *P* < 0.001). “Somatization subscale from SCL-90R” explained 9% of the variance and “years on methadone treatment” accounted for an additional 4% of the variance of AUDIT scores (*β* coefficient 0.36 *P* = 0.023).

## 4. Discussion

The results from the present study are largely consistent with findings from other studies [58, 59] that assessed current levels of general psychopathology with SCL-90R as a diagnostic screening tool, suggesting that patients on methadone maintenance treatment have high rates of psychopathological symptoms and female patients are in particular distress [60–62]. According to a recent article [63], among the demographic variables, age significantly differentiated patients, as sensitive-psychotic, violent/suicidal, and panic addicts proved to be younger, whereas older heroin addicts were distinguished by higher scores for somatization and worthlessness. Our results reporting age being negatively correlated with anxiety, paranoid ideation, phobic anxiety, hostility, and interpersonal sensitivity subscales are in agreement with the study mentioned above.

Yet, unlike other reports [59, 64], our sample was characterized by remarkably high levels of unemployment (81.53%), possibly reflecting the lack of social supportive systems in our country and social stigma along with high rates of psychopathology evidenced in these people (only 6.4% of our sample scored within normal range on SCL-90R subscales) rendering them unable to claim a position in the marketplace.

The fact that the largest proportion of our sample reported being unmarried or divorced and without any children highlights some of the social consequences of addiction, affecting their family environment [65]. Earlier reports state that addicted individuals with higher social adjustment (i.e. married, better educated, and employed) had lower risk of relapse to daily illicit drug use [66, 67]. In our sample in agreement with other findings [39, 68] single patients had higher average scores on all domains of psychopathology compared to married ones, however, with no significant discrepancy. Divorced or separated subjects exhibited higher scores on AUDIT scale possibly reflecting the role of interpersonal determinants in alcohol abuse [15, 69].

Further studies [58, 59] ranked depressive symptomatology first among methadone patients in treatment and reported [58, 59, 68] significantly higher prevalence of anxiety and hostility in female methadone maintenance treatment patients compared to males. In terms of drug abuse duration, a fairly large proportion of participants in our study reported having a history of addiction since before the age of 15, necessitating the implementation of prevention strategies among young people [70, 71]. However, in agreement with other authors [63, 68] drug abuse duration does not have significant influence on psychopathology scales, as if a kind of adaptability develops.

Coexisting psychopathology substantially affects quality of life [72, 73] and authors recommend [29, 74] that self-reported psychopathology should be routinely evaluated in order to improve health-related quality of life in comprehensive treatment programs for heroin abusers.

The relationship between addictive disorders and other forms of psychopathology has long been debated since it may involve such a close interaction at neurobiological levels between predisposing factors, addictive processes, and addictive consequences that the attempt to clinically distinguish between addictive-related and independent psychopathological symptoms may turn out to be little more than an inconclusive theoretical exercise. This is the reason that the application of the classic model of psychiatric comorbidity in the field of addiction has been the target of criticism owing to the difficulty of disentangling supposedly independent psychiatric symptoms and syndromes from the core psychopathology of addiction [75–77].

Another important issue is the prevalence of alcohol abuse and dependence which is increasingly acknowledged [12, 78–82] in methadone maintenance treatment settings, adversely affecting their program compliance and physical and mental health. Among the variables associated with alcohol use by methadone patients is psychiatric symptomatology. Alcoholics scored higher [78] than nonalcoholics on six subscales of the Brief Symptom Inventory (BSI): somatization, obsessions-compulsions behavior, depression, anxiety, phobic anxiety, and psychotic symptoms, but authors comment that because the findings are correlational, reported psychopathology might be an alcohol-induced artifact that is secondary to dependence.

In the predictive model of our study, 9% of the variance of scores on AUDIT was attributed to somatization subscale from SCL-90R and years on methadone treatment explained an additional 4% of the variance. Somatization in drug addicts is distinguished by a number of somatic and anxious elements, which are usually a feature of opiate withdrawal. In the late 90s it was proposed that alcohol abuse during methadone maintenance treatment might result from inadequate methadone dose [11] or that chronic alcohol use increases CYP450 3A4 metabolism of methadone resulting in the need for increasing dose being required to sustain effective methadone serum levels. Alcohol abuse was alleged to occur when dosage is likely sufficient to control most withdrawal symptoms and block opioid-related euphoria, due to cross tolerance, but is not sufficient to control drug craving. Witkiewitz [83] stated that proximal risk factors, such as craving, mood, and stress, are associated with distal risk factors, such as psychological distress, possibly leading to continued substance and alcohol use. The theory was that patients initiate or increase their use of alcohol in order to achieve a change in their moods or function that was no longer accessible to them through opioid abuse, providing an alternative rewarding effect. Also since daily urinalyses informed about their actual heroin use status in order to remain on methadone maintenance, a condition is brought about in which abuse of alcohol becomes more rewarding than opioid abuse.

Results from other studies [16, 84, 85] indicate that drinking problems among patients undergoing methadone maintenance treatment are associated with an increased risk of relapse into illicit drug use and with discharge from treatment. Authors suggest that concurrent treatment of alcohol-related problems including systematic monitoring of alcohol use should be recommended [86–88] to reduce the risk for relapse into illicit drug use and improve overall treatment outcome in methadone maintenance treatment settings, especially among patients with personality disorders [89–91].

In sum, our study indicates the presence of high rates of psychopathological symptoms along with problematic alcohol use among drug addicts that hinders the positive outcome of therapeutic efforts in substitution programs. This should be taken into account during the therapeutic treatment process, so as to implement the most effective and most intensive interventions [92–94].

## 5. Limitations 

The limited number of participants compared with the total number of drug addicts treated in all Substitution Units of our country may compromise the generalizability of our findings and additional extended surveys are required to conclude more accurately.

It is also important to emphasize that the results of the present study evidence psychiatric symptomatology and not psychiatric diagnosis among drug addicts on methadone maintenance treatment owing to lack of necessary diagnostic tools but beyond this owing primarily to lack of information about concurrent substance abuse among participants leading them to experience relevant intoxication or withdrawal symptoms which might have affected their answers in applied questionnaires.

## 6. Conclusions

High levels of psychological distress along with high prevalence of alcohol-related problems are evidenced among drug addicts on Methadone Substitution Treatment Programs, in Greece. Moreover the fact that our research was conducted in a time of economic crisis, with limited work opportunities, provides a further explanation for the high unemployment rates in our sample. Targeting ancillary psychosocial services [95–97] which focus on legal, educational, vocational, recreational, financial, and family issues, as well as interpersonal difficulties, would be beneficial for this population. These factors should all be addressed in order to provide a more flexible approach and improved delivery of these needed, life-saving services.

## Figures and Tables

**Table 1:** Mean scores on SCL-90R subscales and on AUDIT scale.

	Total	*N*	Male *N* = 98	*N*	Female *N* = 32	*N*	*t*-test *P*
Age	43.34 ± 8.12	130	44.3 ± 8.30	98	40.4 ± 8.24	32	0.010
Years of addiction	26.07 ± 7.93	130	27.0 ± 7.67	98	23.5 ± 8.17	32	0.021
Depression	2.00 ± 0.86	126	1.90 ± 0.84	95	2.33 ± 0.84	31	0.016
Obsessions-compulsions	1.89 ± 0.88	126	1.80 ± 0.88	95	2.15 ± 0.85	31	0.052
Paranoid ideation	1.73 ± 0.89	126	1.66 ± 0.90	95	1.95 ± 0.84	31	0.109
Anxiety	1.56 ± 0.95	125	1.43 ± 0.88	94	2.00 ± 1.06	31	0.003
Somatization	1.40 ± 0.88	127	1.30 ± 0.81	95	1.70 ± 1.02	32	0.024
Hostility	1.39 ± 1.05	126	1.26 ± 0.99	95	1.81 ± 1.13	31	0.011
Interpersonal sensitivity	1.29 ± 0.80	126	1.27 ± 0.78	95	1.38 ± 0.86	31	0.505
Psychoticism	1.21 ± 0.76	126	1.16 ± 0.73	95	1.37 ± 0.87	31	0.191
Phobic anxiety	0.77 ± 0.77	126	0.68 ± 0.71	95	1.06 ± 0.89	31	0.015
AUDIT	6.91 ± 7.96	127	6.59 ± 7.80	96	7.90 ± 9.03	31	0.428

**Table 2:** Percentages of pathological values in SCL-90R and AUDIT.

SCL-90R						
	*N*	Raw scores				*T* score *T* ≥ 70
		0-1	1^+^-2	2^+^-3	3^+^-4	
Somatization	127	39.4%	39.3%	15.8%	5.5%	29.2%
Obsessions-compulsions	126	18.3%	38.8%	33.4%	9.5%	34.9%
Interpersonal sensitivity	126	39.7%	43.6%	14.6%	2.3%	11.9%
Depression	126	15.9%	33.3%	38.9%	11.9%	42.9%
Anxiety	125	34.4%	38.4%	18.4%	8.4%	27.2%
Hostility	126	42.9%	32.5%	16.7%	7.9%	15.1%
Phobic anxiety	126	71.4%	19.1%	9.2%	0.0%	19%
Paranoid ideation	126	25.4%	42.1%	24.6%	7.9%	22.2%
Psychoticism	126	42.1%	45.0%	9.7%	5.2%	15.1%

AUDIT (*N* = 127)						

0–7		63%				
Indication of alcohol abuse (AUDIT 8–15)		24.4%				
Serious abuse/addiction (AUDIT > 15)		12.6%				

**Table 3:** Correlations among scores on SCL-90R subscales and AUDIT scale as to age, education level, years of abuse, and time on methadone treatment.

		Age	Educ.	Years of abuse	Y. Meth.	Som.	O. C.	In. Sen.	Dep.	Anx.	Host.	Ph. An.	P. I.	Psych.
Years of education (Educ.)	*r*	0.071												
	*P*	0.420												
Years of abuse	*r*	0.790	−0.018											
	*P*	0.000	0.840											
Years of methadone (Y. Meth.)	*r*	0.237	0.044	0.264										
	*P*	0.008	0.630	0.003										
Somatization (Som.)	*r*	−0.141	−0.091	0.018	−0.045									
	*P*	0.114	0.306	0.837	0.623									
Obsessions-compulsions (O. C.)	*r*	−0.175	−0.060	−0.005	0.002	0.738								
	*P*	0.050	0.503	0.952	0.984	0.000								
Interpersonal sensitivity (In. Sen.)	*r*	−0.278	−0.005	−0.158	−0.102	0.538	0.651							
	*P*	0.002	0.954	0.078	0.266	0.000	0.000							
Depression (Dep.)	*r*	−0.143	−0.038	−0.036	0.010	0.712	0.795	0.688						
	*P*	0.110	0.673	0.686	0.917	0.000	0.000	0.000						
Anxiety (Anx.)	*r*	−0.224	−0.078	−0.055	−0.061	0.787	0.806	0.688	0.821					
	*P*	0.012	0.386	0.545	0.506	0.000	0.000	0.000	0.000					
Hostility (Host.)	*r*	−0.262	−0.033	−0.067	−0.076	0.535	0.598	0.626	0.618	0.632				
	*P*	0.003	0.717	0.457	0.405	0.000	0.000	0.000	0.000	0.000				
Phobic anxiety (Ph. An.)	*r*	−0.201	0.030	−0.106	−0.110	0.580	0.610	0.588	0.618	0.747	0.431			
	*P*	0.024	0.739	0.237	0.230	0.000	0.000	0.000	0.000	0.000	0.000			
Paranoid ideation (P. I.)	*r*	−0.261	−0.081	−0.049	−0.205	0.646	0.729	0.687	0.649	0.721	0.622	0.555		
	*P*	0.003	0.364	0.583	0.024	0.000	0.000	0.000	0.000	0.000	0.000	0.000		
Psychoticism (Psych.)	*r*	−0.146	−0.031	−0.029	−0.097	0.689	0.742	0.790	0.745	0.789	0.620	0.660	0.748	
	*P*	0.102	0.731	0.747	0.288	0.000	0.000	0.000	0.000	0.000	0.000	0.000	0.000	
AUDIT	*r*	−0.034	−0.092	0.106	0.187	0.341	0.247	0.230	0.262	0.347	0.233	0.099	0.302	0.292
	*P*	0.701	0.304	0.237	0.039	0.000	0.005	0.010	0.003	0.000	0.009	0.272	0.001	0.001
